# Combination of a MIP3α-antigen fusion therapeutic DNA vaccine with treatments of IFNα and 5-Aza-2’Deoxycytidine enhances activated effector CD8+ T cells expressing CD11c in the B16F10 melanoma model

**DOI:** 10.21203/rs.3.rs-3243336/v1

**Published:** 2023-08-14

**Authors:** Kaitlyn Fessler, James T. Gordy, Avinaash K. Sandhu, Yinan Hui, Aakanksha R. Kapoor, Samuel K. Ayeh, Styliani Karanika, Petros C. Karakousis, Richard B. Markham

**Affiliations:** Johns Hopkins Bloomberg School of Public Health; Johns Hopkins Bloomberg School of Public Health; Johns Hopkins Bloomberg School of Public Health; Johns Hopkins Bloomberg School of Public Health; The Johns Hopkins Hospital; The Johns Hopkins Hospital; The Johns Hopkins Hospital; The Johns Hopkins Hospital; Johns Hopkins Bloomberg School of Public Health

**Keywords:** MIP3α/CCL20, IFNα/Interferon alpha/Type I interferon, 5-Aza-2’-Deoxycytidine/Decitabine/Dacogen, CD11c, Tumor microenvironment, CD11c+ CD8+ T cell, DNA Therapeutic Cancer Vaccine

## Abstract

Previous studies in the B16F10 mouse melanoma model have demonstrated that combining a DNA vaccine comprised of regions of gp100 and tyrosinase-related protein 2 fused to Macrophage-inflammatory protein 3-alpha (MIP3α) with recombinant Interferon alpha (IFN) and 5-Aza-2’-Deoxycytidine (5Aza) treatments resulted in significantly greater anti-tumor activity and immunogenicity in the tumor microenvironment (TME). This brief report details that the combination of vaccine with treatments IFN and 5Aza results in both the upregulation of genes expressing CD11c-interacting proteins and an increase in the TME of a distinct CD11c+ CD8+ T cell population. This cell population correlates with tumor size, is primarily comprised of effector or effector memory T cells, and has a more robust response to ex vivo stimulation as compared to CD11c− CD8+ T cells as measured by surface activation markers 4-1BB (CD137) and KLRG1 (Killer cell lectin-like receptor G1) and intracellular IFNγ production. In conclusion, this combination therapy results in greater presence of highly active effector CD8+ T-cells expressing CD11c in the TME that correlate with and are likely primary contributors to treatment efficacy.

## Introduction

**Figure 1:**
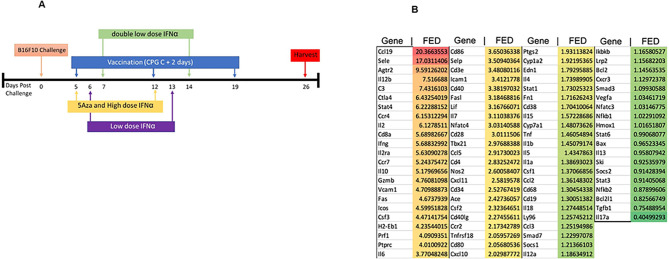
Therapy Timeline and Genetic Expression of a Panel of Mouse Immune Genes. A) Timeline for treatments for all experiments in this study. B) Genetic expression was compared between pooled groups (n=5) of vaccine alone and vaccine+IFN+5Aza. Genes were normalized to the GAPDH gene to create a ΔCt value and then compared to each other to create a ΔΔCt value. Fold Expression Difference (FED) was calculated by 2^−ΔΔCt^.

Cancer therapeutic vaccines combined with immunotherapies and chemotherapies have garnered interest due to their potential synergistic effects[1]. Previous preclinical studies have demonstrated that DNA vaccines comprising melanoma antigen(s) fused to MIP3α delayed tumor growth, prolonged survival, and enhanced T-cell immunity in the B16F10 melanoma model[2–4]. MIP3α vaccine fusions have been shown to target antigen(s) to immature dendritic cells (iDC), with presentation through MHC class I and II[5, 6]. Here, antigens gp100_25–235_ and tyrosinase-related protein 2 (Trp2_170–269_) that have shown relevance in the clinic[7] are fused to MIP3α. When MIP3α-Gp100-Trp2 vaccine adjuvanted with delayed administration of CpG type C (henceforth referred to as “vaccine”) was combined with the drug treatments low-dose 5Aza-2’Deoxycytidine (5Aza) and a series of high and low doses of recombinant IFNα (IFN) (Fig. 1A), significantly greater anti-tumor activity was achieved, including increased dendritic cell (DC) and CD8 + T-cell infiltration into the tumor microenvironment (TME)[4, 8].

CD11c is a type I transmembrane protein forming part of the complement receptor 4 and has been shown to play a role in phagocytosis, cell migration, cytokine production, and T-cell proliferation[9]. CD11c binds to adhesion molecules, such as intercellular adhesion molecule 1 (ICAM1) and vascular cell adhesion molecule 1 (VCAM1), as well as the complement component 3 (C3) degradation product iC3b[10]. CD11c has been shown to be expressed on most DCs and some B cells, macrophages, and monocytes[11]. A subset of CD8 + T cells expressing CD11c + have been described in various tissues, infections, and cancers[9, 12–14] with the ability to become suppressor or effector cells[15–18].

The current study shows that CD8 + T cells expressing CD11c are enriched in the TME with combination treatment and correlated with tumor size in vaccinated groups, To our knowledge, this study is the first to show the presence of CD8 + CD11c + T cells in a tumor-associated antigen based therapeutic cancer vaccine system, and the data suggest that these cells are in higher numbers, are more robust, and are more potent with the combination treatments compared to vaccine alone or treatments alone. These cells are therefore likely to be critical components in the tumor suppression observed with the therapeutic regimen we have employed against the B16F10 melanoma.

## Materials and Methods

### Animals

6–12-week-old female C57BL/6 (Charles River, Wilmington, MA) mice were challenged with a lethal dose of B16F10 melanoma (5×10^4^ cells, > 95% viability) administered intradermally on the mouse flank on day 0 of therapy[2–4, 8]. Tumor size was recorded by calipers every 1 – 3 days as square mm (L × W). Mice were monitored for signs of distress in accordance with IACUC protocols and were removed from the study once tumor diameter exceeded 1 cm, if the tumor was ulcerated and bleeding, or if mice showed signs of distress.

### Vaccines and Therapies

Vaccine antigen is the MIP-3α-Gp100-Trp2 (tyrosinase-related protein 2) DNA construct in the pCMVe mammalian expression plasmid published here[8]. Therapy including vaccine, recombinant mouse interferon alpha-A (IFNα, R&D Systems, Inc. Minneapolis, MN), and InSolution^™^ 5 Aza 2’-deoxycytidine (5Aza, CalBiochem^®^, MilliporeSigma, Burlington, MA) has been detailed in prior publications[8] and is outlined in Fig. 1 A.

### Tumor Lysate qRT-PCR

Cross-sections of tumor weighing less than 100 mg were harvested. Total RNA was extracted from tumors by Trizol^®^ (Ambion^®^ by Life Technologies, Carlsbad, Ca), with homogenization performed by the Fisher Scientific^™^ PowerGen125 (Thermo Fisher Scientific, Waltham, MA). RNA from five mouse samples in the group were equally pooled. The cDNA reverse transcription reaction utilized the SuperScript^™^ III First-Strand Synthesis System (Invitrogen ^™^, Waltham, MA). Real-time quantitative reverse transcription-PCR (qRT-PCR) was performed utilizing TaqMan^®^ Gene Expression Fast Advanced Master Mix and the TaqMan^®^Gene Expression Array: Mouse Immune Panel (Applied Biosystems^™^, Halethorpe, MD). The pooled group cDNA was equally loaded into the wells of the array plate. Cycle threshold (Ct) was normalized to GAPDH to calculate ΔCt. The ΔCt values across the experimental conditions of mice vaccinated alone or vaccinated and treated with IFN + 5Aza were subtracted to create a ΔΔCt statistic. Fold Expression Difference (FED) was calculated by the formula 2^^^(−ΔΔCt). qRT-PCR was performed utilizing the StepOnePlus^™^ machine and software (Applied Biosystems^™^ by Thermo Fisher, Halethorpe, MD).

### Flow Cytometry

Tumor cell and splenic suspensions were prepared as previously described, with or without the Lympholyte M (Cedarlane Labs, Burlington, NC) purification step[3, 4]. Briefly, tissue was extracted, kept cold, ground through a filter, washed, either purified or processed to lyse red blood cells, and used for downstream applications. Splenocytes were cryopreserved in 90% FBS 10% DMSO freezing media using isopropanol baths at −80°C prior to moving the samples to −150°C. Splenocytes were quick-thawed and allowed to rest for at least 2 hours at 37°C. Tumor cell suspensions were processed the same day as harvest. Cells were stained in a 96-well V-bottom plate (Sarstedt, Inc., Newton, NC), with combinations of the anti-mouse mAbs described in Supplementary Table 1. The Attune^™^ NxT (Thermo Fisher Scientific, Waltham, MA) flow cytometer was utilized. Flow data were analyzed by FlowJo Software (FlowJo, LLC Ashland, OR) or Attune NxT Software v3.2.1. Tumors smaller than 25mm^2^ were not analyzed due to an insufficient amount of tissue. Supplemental Fig. 1 outlines gating structure and stimulation procedures[4]. Gates were formulated using full-minus-one (FMO) staining controls as reference.

### Statistics

Datasets comparing three groups were tested by one-way ANOVA with Tukey’s or with Dunn’s multiple comparison test based on D’Agostino & Pearson normality test. Datasets comparing two groups were analyzed by Mann-Whitney tests. Scatter plots were analyzed by linear regression with Pearson correlation coefficient test. Grouped experiments were analyzed by 2-way ANOVA with Sidak’s multiple comparison test. Sample sizes are defined in the figure legends. GraphPad Prism^™^ 10 (GraphPad Software, Inc. San Diego, CA) and Microsoft^®^ Excel^™^ (Microsoft, Corp., Redmond, WA) were utilized for statistical analyses and figure creation. Error bars represent the estimation of the standard error of the mean and midlines the group mean. α ≤ 0.05.

## Results

### Tumor Lysate Expression of CD11c Ligands

Previous studies have shown the upregulation of chemokines CCL19, CCR7, XCL1, and CXCL10 in the tumor lysate of mice treated with the combination therapy outlined in Fig. 1A[4, 8]. A qRT-PCR panel was performed to elucidate the change in expression of additional immune-related genes in the pooled tumor lysates between the groups receiving vaccine alone and combination therapy. The panel verified that CCL19, CCR7, and CXCL10 were all upregulated. With addition of IFN and 5Aza, T-cell-related inflammatory genes were upregulated in the panel more than 4-fold, such as IFNγ, STAT4, IL12b, IL2, IL2ra, GranzymeB, Fas, and others (Fig. 1B). Interestingly, adhesion markers, such as VCAM1 (4.7-fold) and ICAM1 (3.4-fold), were upregulated, as was C3 (7.4-fold) (Fig. 1B). Our previous work showed enhanced infiltration of CD11c + DC[8], and we hypothesized that other cell types such as T cells may also be expressing CD11c.

### CD8 + 11c + T cells in the TME

**Figure 2:**
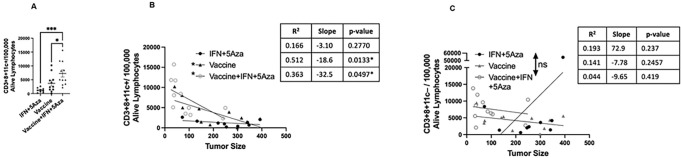
CD3+CD8+CD11c+ cells in the TME. A) The number of CD3+CD8+CD11c+ cells per 100,000 alive lymphocytes between treatment groups. B) The correlation of CD3+CD8+CD11c+ cells and C) CD3+CD8+CD11c− cells to tumor size stratified by group. D) Across treatment groups, CD11c+ and CD11c− CD8 T cells were analyzed for markers of effector state. CD44+CD62L+ are Tcm, CD44+CD62L-are Tem/Teff, and CD44-CD62L+ are Tnaive. These data were combined from three independent experiments: with sample size ranging from 9–11 total samples per group *p<0.05, **p<0.01, ***p<0.001.

A subset of CD8 + T cells expressing the marker CD11c, have been described in the TME aiding in tumor suppression[12]. To analyze whether if these cells are present in this system, flow cytometric analysis was performed on tumor cell suspensions from mice treated with the combination therapy, vaccine alone, or with IFN + 5Aza alone. The CD8 + CD11c + T-cell population was significantly higher following combination therapy vs. vaccine alone (p = 0.0271) or IFN + 5Aza (p = 0.0007) (Fig. 2A). Of note, when stratified across groups, the presence of the CD11c + CD8 + T-cell subpopulation was also correlated with tumor size in vaccinated groups, whereas CD11c− CD8 + T cell numbers were not correlated (Fig. 2B–C).

### Effector Phenotype of CD8 + 11c + T cells in the TME

The functionality of CD3 + CD8 + CD11c + cells have been assessed in disparate model systems, with dramatically different results[9, 12–14]. Therefore, in this study, the overall phenotype of this population infiltrating the TME was explored, independent of treatment group. CD3 + CD8 + CD11c + cells were categorized into three groups based on CD44 and CD62L expression: CD62L + CD44 + central memory (Tcm); CD62L-CD44 + effector memory (Tem); and CD62L + CD44− naive (Tnaive). CD8 + CD11c + cells had a significantly greater proportion of Tem cells compared to their CD11c− counterparts (p = 0.01) (Fig. 2D). Interestingly, the CD8 + CD11c− T cells had significantly greater proportions of Tnaive (p < 0.001) and Tcm (p = 0.001) cells compared to the Cd8 + CD11c + T cells (Fig. 2D).

### Activation of CD8 + 11c + T cells in the Spleen

**Figure 3:**
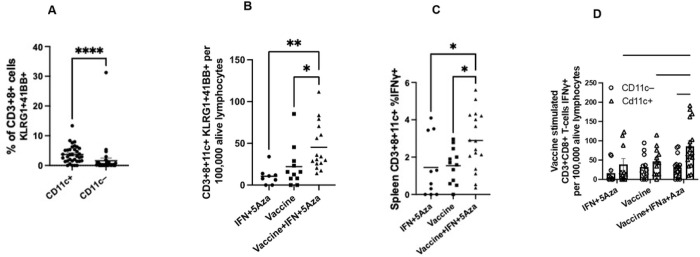
Stimulation of Splenic T cells From Vaccinated Mice: At day 26 of the standard therapeutic mouse protocol, spleens were harvested and cryopreserved. Thawed splenocytes were rested at 37°C for at least 2 hours and then were stimulated with vaccine peptides for four hours in the presence (IFNy) or in the absence (KLRG1/41BB) of cell transport inhibitors, and then assayed by flow cytometry. A) Including all groups, percentage of CD11c+ or CD11c− CD8 T-cells expressing KLRG1 and 41BB. B) The number of KLRG1+41BB+ CD11c+ CD8 T cells post-stimulation across the groups. C) Percentage across groups of CD11c+ CD8+ T cells expressing IFNy post-stimulation. D) Number of CD11c+ and CD11c− CD8 T cells expressing IFNy post-stimulation across the groups and stratified by CD11c positivity. Samples are derived from three independent experiments with sample size ranging from 8–17 total samples per group. *p<0.05, **p<0.01, ****p<0.0001.

Cryopreserved splenocytes from the same studies as the above TME data were analyzed to further assess the subset of CD11c + CD8 + T cells. Since expression of 4-1BB, a costimulatory marker, and Killer cell lectin-like receptor G1 (KLRG1), a marker of antigen-experienced cells, have been described in the literature to be expressed on the CD8 + CD11c + T-cell population[12], these markers were assessed following ex-vivo stimulation with vaccine peptides. Co-expression of KLRG1 and 4-1BB was significantly higher in CD8 + CD11c + T cells (p< 0.001) compared to their CD11c– counterparts (Fig. 3A). Further, when stratified by treatment group, expression of 41BB and KLRG1 on the surface of CD8 + CD11c + T cells was increased significantly following combination therapy relative to treatment with IFN + 5Aza (p = 0020) or vaccine alone (p = 0.0250) (Fig. 3B).

Additionally, the functionality of CD3 + CD8 + CD11c + splenocytes across groups was investigated by measuring IFNγ production after ex-vivo stimulation with vaccine peptides in the presence of cell transport inhibitors using intracellular staining flow cytometry methods. IFNγ expression was statistically significantly different between the combination group and IFN + 5Aza group (p = 0.0393) and the vaccine group (p = 0.0426) (Fig. 3C). Additionally, after stratifying the CD8 + T cells by CD11c expression, the CD11c + population had a statistically significantly increased proportion of cells expressing IFNγ between the combination group and the IFNα and 5Aza group (p = 0.0110) and the vaccine group (p = 0.0319) (Fig. 3D). Moreover, when compared to the CD11c− population in the combination group, the CD11c + cells had a significantly greater proportion of cells expressing IFNγ (p = 0.0019) (Fig. 3D).

## Discussion

Previous studies in our laboratory have shown that the antitumoral efficacy of a MIP3α-fused vaccine targeting gp100 and trp2 was enhanced with the combination of 5Aza and IFN, resulting in increased chemokine expression and infiltration of immune cells into the TME[4, 8]. The current study provides evidence that the combination therapy additionally induces the upregulation of inflammatory and especially T-cell-related genes, as well as the CD11c ligands ICAM1, VCAM1, and C3 (Fig. 1). Additionally, this study elucidates that CD8 + CD11c + T cells are increased with combination therapy and are correlated with tumor size, while the CD11c− CD8 + T cells are not (Fig. 2).

The majority of the CD8 + CD11c + T cells consisted of Tem cells, with fewer Tcm and naive T cells compared to the CD11c− T cells (Fig. 2). Following ex vivo stimulation, CD8 + CD11c + T cells elicited by the combination therapy also expressed more of the costimulatory marker 4-1BB, the antigen-experienced marker KLRG1, and the effector cytokine IFNγ compared to those elicited by the vaccine alone or treatment with IFNα and 5Aza (Fig. 3). 4-1BB is hypothesized to be a necessary costimulatory marker for CD8 + CD11c + T cells and is known to enhance cellular immunity by promoting T-cell survival and enhancing cytokines, such as IFNγ, upon antigen presentation[19]. KLRG1 expression is hypothesized to play a role in CD8 + CD11c + T-cell activity and is induced on CD8 + T cells that are highly cytolytic short-lived effectors[20]. Interestingly, the majority of the IFNγ production is due to the CD8 + CD11c + and not the CD8 + CD11c− T-cell population (Fig. 3), suggesting the former is likely a primary contributor to the antitumoral effects seen with combination therapy.

Although some studies have pointed to stronger regulatory activity[9], other studies have shown that this subset of CD8 + T cells expressing CD11c aids in successful antitumoral responses via enhanced effector functions[15–18]. Our results suggest that vaccine-induced, activated CD8 + CD11c + T cells with the capacity to perform effector functions significantly contribute to the antitumoral effect elicited by our combination therapy. The presence of CD8 + CD11c + T cells and their capabilities are dependent on the combination of vaccine and drug treatments, as removing either arm limits the induction of these cells. Studies to further delineate how these T cells arise and function will be essential to understanding the efficacy of the combination therapy and will provide clues for further enhancing the therapeutic potential of this regimen.

## Data Availability

Datasets utilized in the study are supplied in Supplementary File 1. Further information is available the corresponding authors upon reasonable request.

## Abbreviations

MIP3α: Macrophage inflammatory protein 3-alpha (aka CCL20)
IFN: Interferon Alpha
5Aza: 5’-Aza-2’-deoxycytidine
TME: Tumor Microenvironment
iDC: immature dendritic cell
Trp2: Tyrosinase-related protein 2
Tcm and Tem: T cell central and effector memory, respectively
KLRG1: Killer cell lectin-like receptor G1
ICAM1: Intercellular adhesion molecule 1
VCAM1: Vascular cell adhesion molecule 1
iC3b: Degradation product of complement component 3 (C3
